# Association of the *Myostatin* Gene with Obesity, Abdominal Obesity and Low Lean Body Mass and in Non-Diabetic Asian Indians in North India

**DOI:** 10.1371/journal.pone.0040977

**Published:** 2012-08-20

**Authors:** Surya Prakash Bhatt, Priyanka Nigam, Anoop Misra, Randeep Guleria, Kalpana Luthra, S. K. Jain, M. A. Qadar Pasha

**Affiliations:** 1 Department of Medicine, All India Institute of Medical Sciences, New Delhi, India; 2 Department of Pulmonary Medicine and Sleep Disorders, All India Institute of Medical Sciences, New Delhi, India; 3 Department of Biochemistry, All India Institute of Medical Sciences, New Delhi, India; 4 Diabetic Foundation (India) and National Diabetes Obesity and Cholesterol Foundation (N-DOC), New Delhi, India; 5 Fortis C-DOC Center of Excellence for Diabetes, Metabolic Diseases, and Endocrinology, Chirag Enclave, New Delhi, India; 6 Department of Biotechnology, Jamia Hamdard, New Delhi, India; 7 Institute of Genomics and Integrative Biology, Delhi, India; Instituto de Investigación Hospital 12 de Octubre, Spain

## Abstract

**Background:**

To determine the association of the A55T and K153R polymorphisms of the *Myostatin* gene with obesity, abdominal obesity and lean body mass (LBM) in Asian Indians in north India.

**Materials and Methods:**

A total of 335 subjects (238 men and 97 women) were assessed for anthropometry, % body fat (BF), LBM and biochemical parameters. Associations of *Myostatin* gene polymorphisms were evaluated with anthropometric, body composition and biochemical parameters. In A55T polymorphism, BMI (p = 0.04), suprailiac skinfold (p = 0.05), total skinfold (p = 0.008), %BF (p = 0.002) and total fat mass (p = 0.003) were highest and % LBM (p = 0.03) and total LBM (Kg) were lowest (p = 0.04) in subjects with Thr/Thr genotype as compared to other genotypes. Association analysis of K153R polymorphism showed that subjects with R/R genotype had significantly higher BMI (p = 0.05), waist circumference (p = 0.04), %BF (p = 0.04) and total fat mass (p = 0.03), and lower %LBM (p = 0.02) and total LBM [(Kg), (p = 0.04)] as compared to other genotypes. Using a multivariate logistic regression model after adjusting for age and sex, subjects with Thr/Thr genotype of A55T showed high risk for high %BF (OR, 3.92, 95% Cl: 2.61–12.41), truncal subcutaneous adiposity (OR, 2.9, 95% Cl: 1.57–6.60)] and low LBM (OR, 0.64, 95% CI: 0.33–0.89) whereas R/R genotype of K153R showed high risk of obesity (BMI; OR, 3.2, 95% CI: 1.2–12.9; %BF, OR, 3.6, 95% CI: 1.04–12.4), abdominal obesity (OR, 2.12, 95% CI: 2.71–14.23) and low LBM (OR, 0.61, 95% CI: 0.29–0.79).

**Conclusions/Significance:**

We report that variants of *Myostatin* gene predispose to obesity, abdominal obesity and low lean body mass in Asian Indians in north India.

## Introduction

Asian Indians are predisposed to develop the metabolic syndrome and type 2 diabetes (T2DM). Among other factors, a major factor contributing to metabolic risk is abnormal body composition. Asian Indians tend to have more abdominal adipose tissue, less lean body mass (LBM) and higher magnitude of insulin resistance (IR) despite falling in the normal range of body mass index (BMI) [1]. The high value of waist hip ratio in Asian Indians may be due to less lean mass of the hips and greater fat at the levels of waist [2]. Another study showed that Asian Indian men have low muscle mass and 30% more total body fat (BF) than other ethnic groups [3]. Low lean mass is also evident in Asian Indian neonates as compared to white Caucasian neonates [4]. Whether low muscle mass in Asian Indians is related to high IR and metabolic syndrome is not yet been adequately investigated. Finally, the reason for these characteristics of body composition has been often stated as due to the “genetic predisposition”, “ethnic factors”, and “chronic protein deprivation” but remains largely uninvestigated [5].

Muscle is the major target for insulin-stimulated glucose uptake, the key determinant of total body insulin sensitivity. Skeletal muscle is the major constituent of LBM and the major determinant of energy expenditure both at rest and during physical activity [6]. Skeletal muscle IR, due to decreased muscle glycogen synthesis, promotes atherogenic dyslipidemia by diverting energy derived from ingested carbohydrates away from muscle glycogen synthesis into increased hepatic *de novo* lipogenesis. These findings are important for understanding the mechanism by which skeletal muscle IR promotes the development of the IR, the metabolic syndrome and T2DM [7].

Myostatin [growth/differentiation factor-8 (GDF-8)], belongs to the transforming growth factor β super family of secreted proteins that control growth and differentiation [8]. Myostatin is expressed uniquely in human skeletal muscle as a 26 kDa mature glycoprotein and secreted into the plasma [8], [9]. Since the first report of human *Myostatin* gene in 1998 [9], six polymorphisms and one intronic mutation have been identified in the *Myostatin* gene [9], [10], [11].

A few studies are available regarding *Myostatin* gene and its functions in animals and humans. Zhiliang *et al* [12] reported that *Myostatin* gene in chickens not only plays an important role in controlling skeletal muscle growth and differentiation, but also appears to have some regulatory function on adipose tissue. In transgenic animal models, a lack of myostatin appears to reduce age-related sarcopenia and loss of muscle regenerative capacity [13]. In human studies, Ferrel *et al.* [14] studied A55T and K153R allelic variants in 49 Caucasians or African Americans, but did not find any significant impact of these polymorphisms in either races, however, a small effect could not be excluded. A loss-of-function mutation (G378A) in the *Myostatin* gene has been associated with muscle hypertrophy in the humans [15]. Gonzalez *et al* [16] studied 41 nonagenarians (33 women, age range, 90, 97 yrs) for A55T, E164K, I225T, K153R and P198A variants of *Myostatin* gene. According to these authors heterozygosity for the K153R polymorphism did not seem to exert any negative influence on the muscle phenotypes of women but homozygosity may do so. Further, *Myostatin* K153R polymorphism was investigated in 281 non-athletic young adults, where it was shown to be associated with the ability to produce ‘peak’ power during muscle contractions [17]. Overall, the genetic basis of muscle mass, and specifically the *Myostatin* gene, has been sparsely investigated in human beings.

We hypothesized that the A55T and K153R polymorphisms in the *Myostatin* gene may have an association with obesity, abdominal and truncal adiposity and LBM in non-diabetic Asian Indians. In this study, we assessed the polymorphic status of the *Myostatin* gene and its correlation with the measures of generalized and abdominal obesity and LBM in Asian Indians residing in north India.

## Materials and Methods

### Study subjects

**Figure 1:**
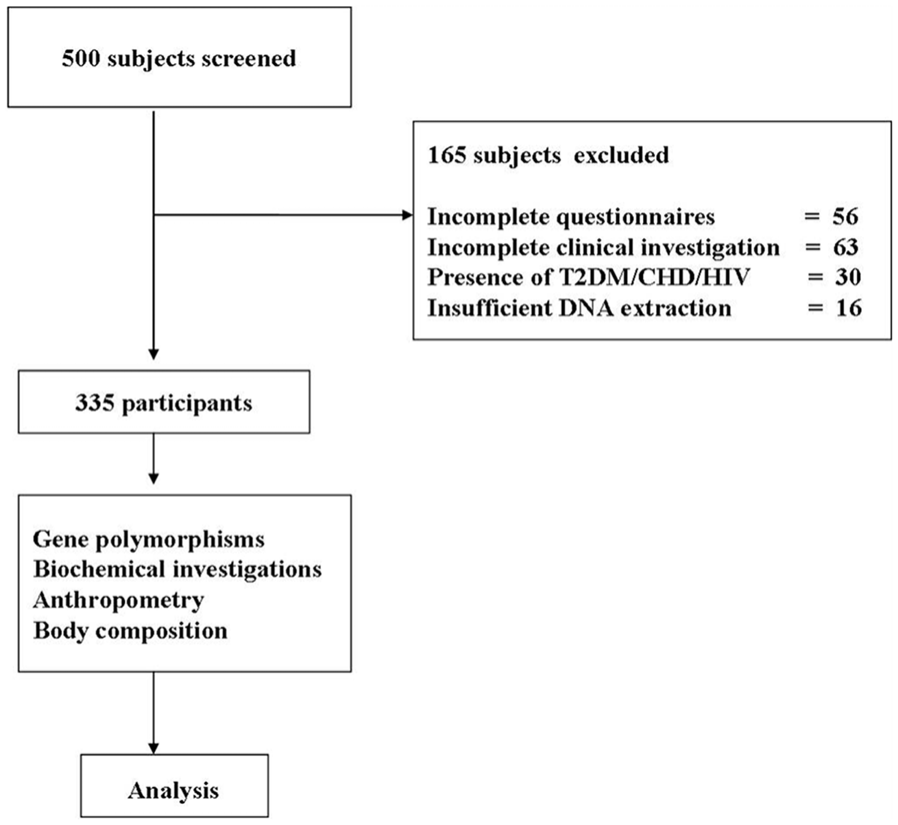
Flow diagram explaining the subject recruitment, selection and reasons for exclusion.

This cross-sectional population-based study was conducted in New Delhi (North India) from May 2006 to October 2011 and was approved by the institute ethics committees of the All India Institute of Medical Sciences, and Fortis hospital, Vasant Kunj, New Delhi, India. A total of 335 subjects (238, males; 97 females) were enrolled in this study after obtaining informed written consent. Subjects were randomly selected from residential colonies to have approximate representation from each income group (high income group∼10%, middle income group∼65–70%, and low income group∼15–20%) according to the proportion living in a metropolitan city. First, a list of total number of houses in each locality with the number of adult subjects in each household was obtained. Subsequently a random number list was generated to select the household that was approached for the participation in the study. Only one individual from one household was selected. Subjects with known T2DM, cardiovascular disease (CVD), severe end organ damage, HIV infection, pregnancy and lactation, were excluded from the study (*figure 1*).

### Clinical and anthropometric measurements

Body weight (to the nearest 0.1 kg) and height (to the nearest 0.1 cm) were recorded without shoes and allowing only light indoor clothes. BMI was calculated using the formula; weight (Kg)/height (m^2^). Circumferences of the waist, hip, mid-thigh, mid arm and neck were recorded to the nearest 0.1 cm. A mean of three readings of each circumference was taken for the calculation of waist-hip ratio. Biceps, triceps, sub-scapular, anterior axillary, supra-iliac, lateral thoracic and thigh skinfolds were measured by using Lange skinfold calipers as described previously [18]. Ratios of sub-scapular and triceps skinfold, and central (sum of sub-scapular and supra-iliac), peripheral skinfolds (sum of biceps and triceps) and sum of all four skinfolds (TSF) were calculated.

### Biochemical analysis

Fasting insulin levels were measured using commercially available radioimmunoassay insulin kits (Immunotech, France) as described previously [19]. The intra- and inter-assay percentage variation was 2.1% and 3.3%.

### Dual Energy X-ray Absorptiometry (DEXA)

Body composition was estimated by DEXA [QDR-2000; Hologic, Waltham, MA, USA] and analyzed using the software version 7.20 as previously described (1, 5). The subject was asked to lie on a padded table, provided with an X-ray generator below the table and a detector above, and was asked to remain still and breathe quietly for a short time while the scanner of the machine passed over the body. The data were collected at 0.5-cm intervals and reported in the standard format. Fat mass (FM), LBM, fat-free mass (FFM) and bone mineral content (BMC) of the whole body and at specific anatomical regions (trunk, arm and legs) were obtained. Total body fat (%BF) was calculated by dividing the FM by the sum of FM, LBM and BMC. Total LBM (%) was calculated by dividing the LBM by the sum of FM, LBM and BMC.

### Genetic analyses

Genomic DNA was extracted from peripheral blood leukocytes by rapid non-enzymatic method [20]. Polymerase chain reaction and restriction fragment length polymorphism of the *Myostatin* gene (K153R) was performed by standard protocols [14]. DNA amplification of the *Myostatin* A55T polymorphism was performed using the forward and reverse primers (New England Biolab, MA, USA) 5′-CCTGTTTATGCTGATTGTTG-3′, 5′-TACTGA GGAT TTGT ACT TAATAG-3′ respectively. The PCR reaction contained 500 ng genomic DNA, 1× PCR buffer, 2.5 µM MgCl2, 200 µM dNTP ((dATP, dCTP, dGTP, and dGTP), 0.2 µM each primer, and 1.25 U Taq DNA polymerase (Sigma, St Louis, MO, USA) in a total volume of 25 µl. Initial denaturation was at 94°C for 5 min followed by 34 subsequent cycles of denaturation at 94°C for 1 minute, annealing at 55°C for 1 minute; extension at 72°C for 1 minute and final extension at 72°C for 10 minutes. The 154 bp PCR (10 µl) product was digested with *AluI* (New England BioLabs, Beverly, MA, USA) restriction enzyme (0.1 µl) at 37°C overnight. The restriction fragments (134 and 20) were electrophoresed in 20% polyacrylamide gels and were visualized using silver staining.

### Statistical analysis

Data were recorded on a pre-designed excel sheet (Microsoft Corp, USA). The allelic and genotypic frequencies were determined by manual counting. Statistical analysis was performed using STATA Version 9 (Stata Corp, Texas, USA). After confirming the normality aspect of quantitative variables, descriptive statistics were computed using Mean ±SD and student *t* test. Difference between proportions was tested using Chi-square test. The influence of the genotype on the clinical parameters was estimated by the Analysis of covariance (ANCOVA) test with multiple comparisons. RFLP of *Myostatin* gene with its respective restriction sites was performed and studied for the genotypes. Allelic and genotypic frequencies were estimated by gene counting, and the observed and expected allele and genotype counts were compared by the Chi-square test to check for Hardy Weinberg Equilibrium. Significant levels for multiple comparisons were corrected by the Bonferroni method. *Myostatin* alleles and genotypes were checked for their association with the clinical, biochemical, anthropometric and body composition profiles. Forward stepwise logistical regression was applied, first adjusting for confounding factors (age, sex and BMI), and to see the independent effect of gene on phenotype variables. Multivariate logistic regression analysis was used for relative risk for obesity (BMI, %BF), abdominal obesity, truncal subcutaneous adiposity and low LBM. The odds ratio (OR) and 95% confidence interval were used as a measure of strength for the association between different A55T and K153R genotypic combinations. Statistical significance was established at a P value of 0.05.

### Definitions

Overweight was defined as BMI≥23 kg/m^2^. WC cut-offs of >90 cm for males and >80 cm for females were considered an indicator of abdominal obesity. Other cutoffs were; FBG≧100 mg/dl, serum TG≧150 mg/dl, blood pressure≧130/85 mmHg and HDL-C; males≤40 mg/dl, and females≤50 mg/dl [21]. IR was measured by two surrogate measures: fasting hyperinsulinemia and HOMA-IR. Hyperinsulinemia was defined as values in the highest quartile as described previously [19]. The value of HOMA denoting IR was termed as HOMA-IR and was calculated as = {fasting insulin ( µU/ml) × fasting glucose (mmol/l)/22·5, [22]}.

## Results

### Clinical, biochemical, anthropometric and body composition profiles (table 1)

**Table 1:** Clinical, biochemical, anthropometric and body composition profiles.

Variables	Total	Males	Females	p-value
Numbers (n)	335	238		97
Age (yrs)	38.0±6.9	38.2±7.0	38.0±6.9	0.2
Body Mass Index (kg/m^2^)	28.5±7.9	27.1±3.2	29.8±3.2	0.02
Insulin (µU/ml)	9.7±3.2	9.6±3.0	7.6±2.3	0.01
HOMA	2.2±1.02	2.2±0.98	1.9±0.8	0.04
Circumferences (cm)				
Waist	92.2±9.6	93.9±9.4	88.8±9.1	0.001
Hip	95.7±7.8	95.1±7.2	97.0±8.2	0.005
Waist hip ratio	0.96±0.1	0.97±0.1	0.86±0.1	0.001
MTC	54.6±8.1	57.2±7.7	48.4±8.4	0.001
MAC	27.8±4.7	27.1± 3.3	28.1±5.7	0.1
Neck	34.3±3.7	35.7 ± 4.2	31.2±3.1	0.01
Skinfolds (mm)				
Biceps	13.1±4.3	13.2±6.1	12.9±5.9	0.3
Triceps	20.6±7.1	18.2±7.0	25.2±7.1	0.001
Subscapular	39.9±10.8	42.8±11.1	33.2±9.7	0.001
Anterior axillary	10.6±3.7	11.0±4.0	8.2±3.4	0.05
Suprailiac	40.0±11.2	43.7±11.3	30.2±9.9	0.001
Thigh	38.9±9.2	39.8±9.3	37.3±8.8	0.05
Lateral thoracic	40.4±11.5	44.1±11.3	30.0±10.6	0.001
Dual Energy X-ray Absorptiometry				
Total body fat (%)	34.7±7.8	35.1±6.7	46.3±8.8	0.002
Total body fat (kg)	26.5±9.6	25.8±7.9	28.2±6.9	0.01
Total lean mass (%)	68.35±12.6	75.20±15.8	54.9±12.8	0.03
Total lean mass (kg)	46.2±8.5	49.4±8.2	39.0±7.7	0.002
Total BMD (g cm^−2^)	1.19±0.14	1.21±0.1	1.10±0 .1	0.001

Values are given as the mean ± standard deviation. HOMA-homoeostasis model assessment for insulin resistance. MTC, mid thigh circumference; MAC, mid arm circumference; BMD, bone mineral density. Statistical significance was established at a P value of 0.05.

The total number of subjects was 335 (238, males; 97 females: mean age: 38.2±7.0 years and 38.0±6.9 years, respectively). There were gender differences in BMI, other body composition parameters, biochemical profile and, insulin and HOMA-IR values.

### Genotype distribution

In A55T polymorphism, the observed frequency of the Ala allele was 0.84 and of the Thr allele was 0.16. Overall, 74.93% subjects were Ala/Ala homozygous, 18.81% were Ala/Thr heterozygous and 6.27% were Thr/Thr homozygous. In K153R polymorphism, the observed frequency of the K allele was 0.84 and of the R allele was 0.16. Overall, 72.84% subjects were K/K homozygous, 22.9% were K/R heterozygous and 5.07% were R/R homozygous. The reproducibility of the genotyping data was checked by 75 duplicate samples.

### Genotype association

Combined data for both genders were analyzed and presented in table 2 for each of the genotypes (Ala/Ala, Ala/Thr and Thr/Thr) of Ala55Thr. BMI (p = 0.04), suprailiac skinfold (p = 0.05), TSF (p = 0.008), %BF (p = 0.002) and total fat mass (p = 0.003) were highest and %LBM (p = 0.03) and total LBM (Kg) were lowest (p = 0.04) in subjects with Thr/Thr genotype as compared to other genotypes. Association analysis of K153R polymorphism (table 3) showed that R/R genotype was associated with significantly higher levels of BMI (p = 0.05), WC (p = 0.04), %BF (p = 0.04), total fat mass (p = 0.03), %LBM (p = 0.02) and total LBM (Kg) were lowest (p = 0.04) in subjects with R/R genotype as compared to other genotypes.

**Table 2:** Association of *Myostatin* (A55T) genotypes with clinical, biochemical, anthropometric and body composition profiles.

Parameters	A/A (1) (n = 251)	A/T (2) (n = 63)	T/T (3) (n = 21)	p value (1&2)	p value (2&3)	p value (1&3)	p value (1, 2&3)
Age (yrs)	37.5±8.7	38.5±9.9	37.0±6.8	0.4	0.5	0.6	0.6
Body mass index (kg/m^2^)	26.4±4.4	27.2±3.9	28.3±4.2	0.3	0.7	0.3	0.04
Insulin (µU/ml)	8.7±3.2	8.4±3.0	9.1±2.3	0.7	0.5	0.8	0.2
HOMA	2.1±1.02	2.2±0.98	2.3±0.8	0.7	0.9	0.5	0.5
Circumferences (cm)							
Waist	91.4±12.0	93.4±11.5	94.4±10.8	0.2	0.4	0.4	0.3
Hip	95.2±9.7	96.7±8.7	97.2±7.9	0.8	0.5	0.7	0.3
WH-R	0.96±0.09	0.97±0.08	0.97±0.05	0.6	0.6	0.8	0.3
Mid thigh	54.2±10.4	55.3±9.7	56.4±12.2	0.4	0.7	0.5	0.4
Mid arm	27.1±3.3	27.9±3.3	28.1±5.7	0.9	0.6	0.4	0.3
Neck	35.7±4.2	37.1±3.1	33.2±3.1	0.09	0.08	0.2	0.5
Skinfolds thickness (mm)							
Biceps	17.0±8.1	17.6±9.4	19.4±7.5	0.7	0.4	0.6	0.2
Triceps	20±9.3	21.1±9.1	21.5±6.4	0.6	0.4	0.4	0.2
Subscapular	39.1±8.6	41.2±8.9	40.1±10.2	0.1	0.3	0.4	0.09
Suprailiac	38.2±13.9	41.3±10.3	45.6±11.5	0.1	0.06	0.04	0.05
Lateral thoracic	39.3±11.2	40.4±12.3	41.1±9.5	0.5	0.7	0.9	0.2
TSF	101.1±30.6	104.1±30.9	126.7±24.9	0.06	0.009	0.001	0.008
Dual Energy X-ray Absorptiometry							
Body fat (%)	35.1±6.7	36.3±8.8	45.2±9.8	0.4	0.02	0.001	0.002
Body fat (%)	35.1±6.7	36.3±8.8	45.2±9.8	0.4	0.02	0.001	0.002
Total lean mass (%)	72.2±12.3	65.2±11.8	51.9±10.8	0.02	0.01	0.002	0.03
Total lean mass (kg)	49.6±8.2	46.8±7.7	45.6±9.6	0.6	0.7	0.06	0.04
Total BMD (g cm−2)	1.21±0.15	1.21±0.08	1.23±0.09	0.9	0.6	0.6	0.8

All values are given as mean ± standard deviation; n, number of subjects. Due to multiple comparisons within each genotype, a Bonferroni correction was applied. Statistical significance was established at a P value of 0.05. HOMA-homoeostasis model assessment; WH-R, Waist-to-hip ratio; TSF, total skinfolds (sum of four skinfold thickness); BMD, bone mineral density.

**Table 3:** Association of *Myostatin* (K153R) genotypes with clinical, biochemical, anthropometric and body composition profiles.

Parameters	K/K (1) (n = 244)	K/R (2) (n = 74)	R/R (3) (n = 17)	p value (1&2)	p value (2&3)	p value (1&3)	p value (1, 2&3)
Age (yrs)	37.0±6.9	37.5±8.05	38.5±5.1	0.9	0.7	0.6	0.5
Body mass index (kg/m^2^)	24.4±2.8	25.5±3.9	28.2±4.6	0.7	0.06	0.05	0.05
Insulin (µU/ml)	7.6±4.2	7.7±5.5	8.0±2.7	0.6	0.5	0.8	0.1
HOMA	1.9±1.02	2.4±0.88	2.3±0.8	0.07	0.4	0.08	0.07
Circumferences (cm)							
Waist	90.4±13.0	91.3±9.8	96.4±10.1	0.4	0.05	0.03	0.04
Hip	96.2±9.2	97.2±8.7	98.4±8.1	0.6	0.8	0.4	0.3
WH-R	0.97±0.09	0.98±0.08	0.98±0.05	0.7	0.6	0.4	0.8
Mid thigh	54.2±10.4	55.3±9.7	56.4±12.2	0.3	0.6	0.2	0.4
Mid arm	26.3±3.2	27.3±3.2	28.4±5.6	0.1	0.7	0.7	0.5
Neck	34.7±4.1	36.2±3.1	36.8±3.1	0.9	0.4	0.6	0.7
Skinfolds thickness (mm)							
Biceps	16.9±7.1	17.8±9.4	18.9±7.5	0.6	0.5	0.6	0.9
Triceps	21±8.3	21.4±8.9	21.7±7.4	0.6	0.3	0.7	0.3
Subscapular	39.5±8.7	40.5±8.9	41.7±9.7	0.3	0.6	0.4	0.09
Suprailiac	39.3±11.9	41.3±10.3	41.8±9.5	0.09	0.5	0.07	0.08
Lateral thoracic	39.3±11.2	41.2±12.3	41.8±9.5	0.4	0.7	0.3	0.4
TSF	103.1±23.6	105.1±28.9	107±24.9	0.5	0.1	0.09	0.9
Dual Energy X-ray Absorptiometry							
Body fat (%)	36.4±7.1	38.5±7.9	43.6±8.8	0.4	0.05	0.03	0.04
Body fat (%)	24.9±8.0	26.8±7.3	30.6±7.8	0.3	0.05	0.02	0.03
Total lean mass (%)	75.7±13.4	63.9±11.3	49.5±10.3	0.01	0.04	0.01	0.02
Total lean mass (kg)	51.6±9.2	45.6±7.3	44.4±9.1	0.2	0.2	0.05	0.04
Total BMD (g cm−2)	1.25±0.15	1.26±0.08	1.28±0.1	0.5	0.4	0.6	0.8

All values are given as mean ± standard deviation; n, number of subjects. Due to multiple comparisons within each genotype, a Bonferroni correction was applied. Statistical significance was established at a P value of 0.05. HOMA-homoeostasis model assessment; WH-R, Waist-to-hip ratio; TSF, total skinfolds (sum of four skinfold thickness); BMD, bone mineral density.

### Logistic regression analysis (figure 2)

**Figure 2:**
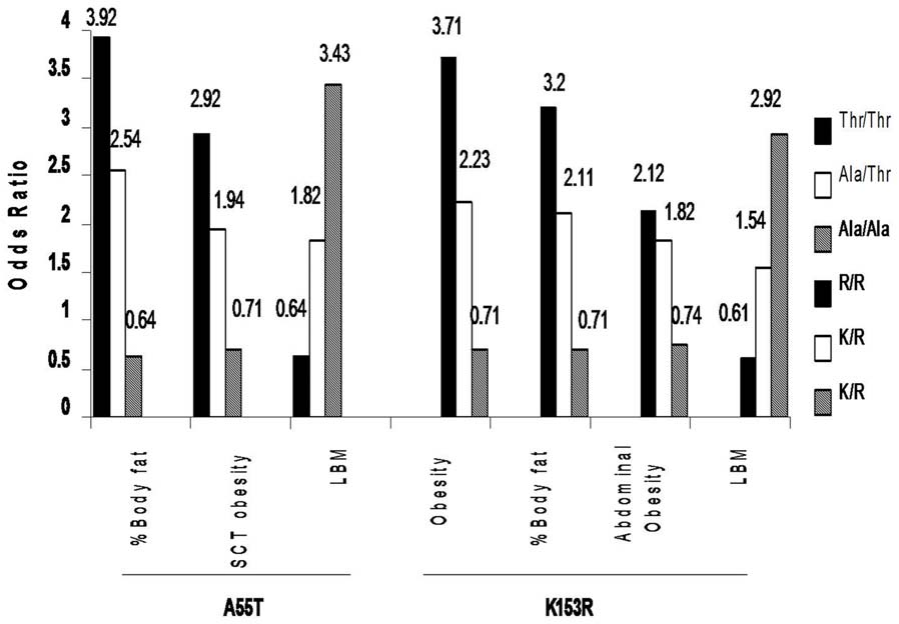
Odds Ratios for obesity, abdominal obesity, subcutaneous adiposity and LBM according to *Myostatin* (A55T & K153R) genotypes. SCT Obesity, subcutaneous obesity; LBM, lean body mass.

Using a multivariate logistic regression model after adjusting for age, sex and BMI, subjects with Thr/Thr genotype of A55T showed high risk for high %BF (OR, 3.92, 95% Cl: 2.61–12.41), truncal subcutaneous adiposity [(based on subscapular skinfold thickness) (OR, 2.9, 95% Cl: 1.57–6.60)] and low LBM (OR, 0.64, 95% Cl: 0.33–0.89) whereas R/R genotype of K153R showed high risk of obesity(BMI; OR, 3.2, 95% CI: 1.2–12.9; %BF, OR, 3.6, 95% CI: 1.04–12.4), abdominal obesity [(based on WC) (OR, 2.12, 95% CI: 2.71–14.23)], and low LBM (OR, 0.61, 95% Cl: 0.29–0.79).

## Discussion

In the present study, K153R and A55T polymorphisms of the *Myostatin* gene was associated with generalized obesity, abdominal obesity and low LBM in non-diabetic Asian Indians. Genetic predisposition to develop excess adiposity and low muscle mass in Asian Indians are important findings, since these may explain the high prevalence of hyperinsulinemia and greater risk for T2DM in this ethnic group [23].

Considering that low muscle mass in Asian Indians may determine insulin resistance, we previously showed that three months of supervised progressive resistance exercises for skeletal muscles significantly improved insulin sensitivity and glycemia and decreased truncal and peripheral subcutaneous adipose tissue in Asian Indians with T2DM [1], [23]. Further, Unni *et al*. [24] have reported that %BF was negatively associated with insulin sensitivity, whereas muscle mass was positively associated with insulin sensitivity in Asian Indian men. Possible reasons for low LBM in Asian Indians remains unclear, and genetic susceptibility remains uninvestigated. Since Asian Indians have been exposed frequently to chronic protein deficiency due to exposure to famines, food shortage, and vegetarian food, it is likely that these factors may have affected the skeletal muscle mass.

The functional role of myostatin in controlling muscle mass has been well documented, however the mechanism by which myostatin controls the muscle fiber number is poorly understood. Myostatin enters the bloodstream as a latent precursor protein and then undergoes a proteolytic process to become a mature peptide that binds to extracellular activin type II receptor (ActRIIB) [25]. Binding of myostatin to ActRIIB induces intracellular activation of Smad proteins; through this pathway, myostatin modulates myoblast proliferation and differentiation [26], and thus ultimately muscle mass. Jiang *et al* [27] showed that the inhibitory domain was located in the region between 42–115 amino acid sequence of myostatin precursor and exerts an important role in the stability of the GDF-8 propeptide inhibitory activity on myostatin activity. Therefore the A55T polymorphism could affect GDF8 activity.

Knowledge about negative regulation of skeletal muscle mass by myostatin was based on loss-of-function models, where myostatin expression and activity was blocked from gestation all throughout life by spontaneous or experimental mutations [26]. The Lys(K)153Arg(R) amino acid replacement was found within the active mature peptide of the myostatin protein and theoretically influenced (a) proteolytic processing with its propeptide (b) affinity to bind with ActRIIB [28]. Reisz *et al*, [29] reported that increased expression of myostatin in skeletal muscle is associated with lower muscle mass and decreased fiber size and myonuclear number, decreased cardiac muscle mass, and increased fat mass in male mice, consistent with its role as an important inhibitor of skeletal muscle mass, in addition to its influences on adipose tissue. Naturally occurring myostatin mutations in cattle have been shown to lead to pronounced hyper muscularity [26].

Data suggest an important role of the myostatin gene in muscle anatomy. McFarlane *et al* [30], suggested that myostatin regulation of postnatal myogenesis involves interactions with numerous downstream signaling mediators (MyoD, via canonical Smad signaling), and notch signaling pathway during inhibition of human myoblast differentiation. Xiao *et al* [31] reported that the negative role of myostatin during muscle regeneration and the increased expression of myostatin observed in Smad3-null muscle may contribute to the regeneration defects. Sartori *et al* [32] showed that the muscle fiber atrophy induced by Smad2/3 activation was prevented by the presence of constitutively active Akt. He also suggested that muscle fibers transfected with a gene encoding dominant negative activin receptor 2B (which inhibits myostatin signaling) are ∼30% larger than normal fibers; this hypertrophy occurs without recruitment of new myonuclei, is diminished by blocking mTOR activity with either rapamycin or mTOR siRNAs, and is enhanced in transgenic mice with constitutively active Akt. Finally, during embryogenesis, myostatin is exclusively expressed in skeletal muscle to control the differentiation and proliferation of the myoblast [8].

Some studies in human beings have suggested that *Myostatin* gene has effects on muscle mass and function. A loss-of-function mutation in the myostatin gene has been associated with muscle hypertrophy in a child [15], Seibert *et al.* [33] reported lower muscle strength (hip and knee flexion and handgrip strength combined) in those who carried the 153R allele in subjects in USA. Santiago *et al.* [17] reported that K153R polymorphism was associated with the ability to produce peak power during muscle contractions, in young non-athletic men in Spain. Han *et al.* [34] reported that higher serum myostatin levels were associated with lower muscle function in subjects from Taiwan. Another study also showed a lower muscle mass/function in a 96 year old woman from Madrid, Spain, with the very rare *Myostatin* R/R genotype compared to her age-matched referents with the 153KK genotype [16]. Further, myostatin expression levels have been shown to be inversely correlated with muscle mass in healthy and HIV-infected subjects [7].

Myostatin may have a strong role in the regulation of adipose tissue mass [35]. Specifically, myostatin-deficient mice have a significant associated to a 70% reduction in fat accumulation [36]. Further, a recent study in mice model suggested that contribution to low fat mass in mice lacking myostatin may be due to increased energy expenditure together with increased leptin sensitivity [37]. It is also possible that the absence of myostatin results in enhanced peripheral tissue fatty acid oxidation and increased thermogenesis, which result in increased fat utilization and reduced adipose tissue mass [35]. Based on our findings, we have a reason to believe that these polymorphisms may have lead to gain of function of myostatin, leading to observed changes in LBM and BF.

### Conclusion

To conclude, we showed association of polymorphisms of *Myostatin* gene with increased adiposity and low LBM in Asian Indians. These findings may have implications for the development of IR and T2DM in Asian Indians, and should be further confirmed in larger studies.
